# Transcriptomic and metabolomic analyses reveal the essential nature of Rab1B in *Toxoplasma gondii*

**DOI:** 10.1186/s13071-023-06030-6

**Published:** 2023-11-08

**Authors:** Kai He, Qiangqiang Wang, Xuwen Gao, Tao Tang, Huiyong Ding, Shaojun Long

**Affiliations:** 1 National Animal Protozoa Laboratory and School of Veterinary Medicine, China Agricultural University, Beijing, 100193 China; 2 National KeyLaboratory of Veterinary Public Health Safety, School of Veterinary Medicine, China Agricultural University, Beijing, 100193 China

**Keywords:** *Toxoplasma gondii*, Transcriptome, Metabolome, Rab family proteins

## Abstract

**Background:**

The protozoan parasite *Toxoplasma gondii* encodes a dozen Rab proteins, which are parts of the small GTPase superfamily and regulate intracellular membrane trafficking. Our previous study showed that depletion of Rab1B caused severe defects regarding parasite growth and morphological structure, yet early defects of endocytic trafficking and vesicle sorting to the rhoptry in *T. gondii* are not expected to have a strong effect. To understand this discrepancy, we performed an integrated analysis at the level of transcriptomics and metabolomics.

**Methods:**

In the study, tetracycline-inducible TATi/Ty-Rab1B parasite line treated with ATc at three different time points (0, 18 and 24 h) was used. We first observed the morphological changes caused by Rab1B depletion via transmission electron technology. Then, high-throughput transcriptome along with non-targeted metabolomics were performed to analyze the RNA expression and metabolite changes in the Rab1B-depleted parasite. The essential nature of Rab1B in the parasite was revealed by the integrated omics approach.

**Results:**

Transmission electron micrographs showed a strong disorganization of endo-membranes in the Rab1B-depleted parasites. Our deep analysis of transcriptome and metabolome identified 2181 and 2374 differentially expressed genes (DEGs) and 30 and 83 differentially expressed metabolites (DEMs) at 18 and 24 h of induction in the tetracycline-inducible parasite line, respectively. These DEGs included key genes associated with crucial organelles that contain the rhoptry, microneme, endoplasmic reticulum and Golgi apparatus. The analysis of qRT-PCR verified some of the key DEGs identified by RNA-Seq, supporting that the key vesicular regulator Rab1B was involved in biogenesis of multiple parasite organelles. Functional enrichment analyses revealed pathways related to central carbon metabolisms and lipid metabolisms, such as the TCA cycle, glycerophospholipid metabolism and fatty acid biosynthesis and elongation. Further correlation analysis of the major DEMs and DEGs supported the role of Rab1B in biogenesis of fatty acids (e.g. myrisoleic acid and oleic acid) (*R* > 0.95 and *P* < 0.05), which was consistent with the scavenging role in biotin via the endocytic process.

**Conclusions:**

Rab1B played an important role in parasite growth and morphology, which was supported by the replication assay and transmission electron microscopy observation. Our multi-omics analyses provided detailed insights into the overall impact on the parasite upon depletion of the protein. These analyses reinforced the role of Rab1B in the endocytic process, which has an impact on fatty acid biogenesis and the TCA cycle. Taken together, these findings contribute to our understanding of a key vesicular regulator, Rab1B, on parasite metabolism and morphological formation in *T. gondii*.

**Graphical Abstract:**

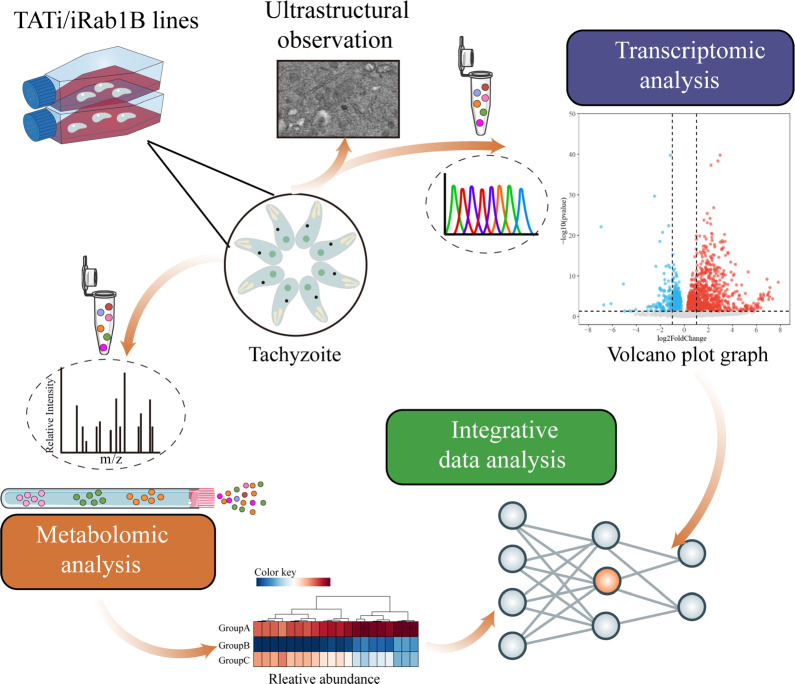

**Supplementary Information:**

The online version contains supplementary material available at 10.1186/s13071-023-06030-6.

## Introduction

*Toxoplasma gondii* is a member of the phylum Apicomplexa, causing toxoplasmosis in a quarter of the human population and almost all warm-blooded animals [1–3]. While healthy individuals may not exhibit obvious symptoms, those with immunodeficiency experience severe symptoms and poor prognosis. For instance, in acquired immunodeficiency syndrome (AIDS) patients, *T. gondii* infection can cause life-threatening encephalitis [4]. Congenital toxoplasmosis occurs when pregnant women are infected for the first time in their life, and its symptoms usually manifest as malformations, visual and intellectual disorders and even abortion [5]. Generally, the establishment of infection is mediated by complex interactions between the parasite and host cell, including invasion processes, parasite replication, nutrient acquisition and evasion of cellular and immunological attacks [6].

As an obligate intracellular parasite, *T. gondii* must scavenge host nutrients to maintain its growth and persistence. In addition to salvaging purine [7], amino acids (tryptophan, arginine, tyrosine) [8], cholesterol [9] and choline [10], *T. gondii* can scavenge host materials including proteins, vitamins, fatty acids and lipids, and it can also partially synthesize fatty acid [10–12], sphingolipid [13, 14], isoprenoids [15] and lipoic acid [16]. After invading host cells, *T. gondii* forms a parasitophorous vacuole (PV) where some molecules enter into the parasite cytoplasm via the specific transporter proteins on the parasite plasma membrane [17]. However, only a few substrates transported via those proteins have been identified. TgNBT1 and TgAT2 were recognized as the high-affinity transporters for nucleobase and nucleoside in *T. gondii* [18]. Apart from the main role of transporting glucose, the *T. gondii* glucose transporter (TgGT1) is capable of transporting galactose, fructose and mannose. ApiAT1 and ApiAT6-1 were responsible for the transport of arginine, and ApiAT5-3 was recognized as the l-tyrosine transporter [19]. TgBT1 was discovered to be a folate transporter and showed high affinity for folic acid [20]. Recently, our study validated the micropore as an essential organelle in the parasite plasma membrane for the endocytic entry point and salvage of nutrients from the host cell cytosol and Golgi [21]. The endocytosed cargos are subsequently delivered to the plant-like vacuole compartment (PLVAC/VAC) via a vesicle-based membrane trafficking process for digestion [22, 23]. Surprisingly, this endocytic process was previously proposed to intersect with the protein sorting to one of the apical organelles, the microneme [24]. Our recent study identified the exact molecules that define the endocytic process, and this study unexpectedly found that one of the key molecules (Rab1B) regulates not only the endocytic vesicles but also the protein sorting to another apical organelle, the rhoptry bulb [23].

Our previous study showed that Rab1B-depleted parasites caused strong defects in parasite replication and morphology. In this study, we combined the multi-omics approaches to understand the molecular effects of the key vesicular regulator Rab1B in the parasite. This detailed information provided insights into the essential nature of Rab1B, reinforcing the combined effect of the key endocytic regulator on the parasite metabolism and cellular morphology. Collectively, our findings gain more evidence to support Rab1B serving as a key regulator of the nutrient salvage process and parasite organelle biogenesis in the zoonotic parasite *T. gondii* and potentially in other related parasites.

## Results

### Depletion of Rab1B resulted in disorganization of the parasite endo-membranes

According to the ToxoDB release v53 database (https://toxodb.org/toxo/), Rab1B appears to have a critical role in parasite growth fitness, as suggested by the low phenotype score in a genome-wide CRISPR/Cas library screen (-5.27). Bioinformatic analysis showed that the key regulator Rab1B is typically a small protein with a molecular weight of 22.8 kDa, which contains a Ras small GTPase domain. To observe the endo-membranes of the parasites depleted with Rab1B, we performed transmission electron microscopy (TEM) on the TATi/Ty-Rab1B (iRab1B) parasites grown in the absence or presence of the inducer anhytrotetracycline (1 μM) (18 and 24 h). In the non-induced parasites, the endomembrane system consisted of typical membranous stacks of Glogi, endosome-like vesicles, the apicoplast and apical organelles (e.g. the micronemes and rhoptry), which are located anterior to the nucleus (Additional file 1: Figure S1a). Compared with the normal organization of the endo-membranes in the non-induced parasites, the endo-membranes in the induced TATi/Rab1B parasites were disorganized with lucent vesicles located anterior to the nucleus and disappearance of many elaborate membranous stacks (Additional file 1: Figure S1b). Parasites with normal endo-membranes and disorganized endo-membranes were quantified, of which up to 30% of the parasites clearly showed disorganized endo-membranes at 24 h of induction (Additional file 1: Figure S1c). This TEM result is consistent with observation of the organelles stained by some specific markers in our previous study [23].

### Transcriptomic analysis and data quality control

**Fig. 1:**
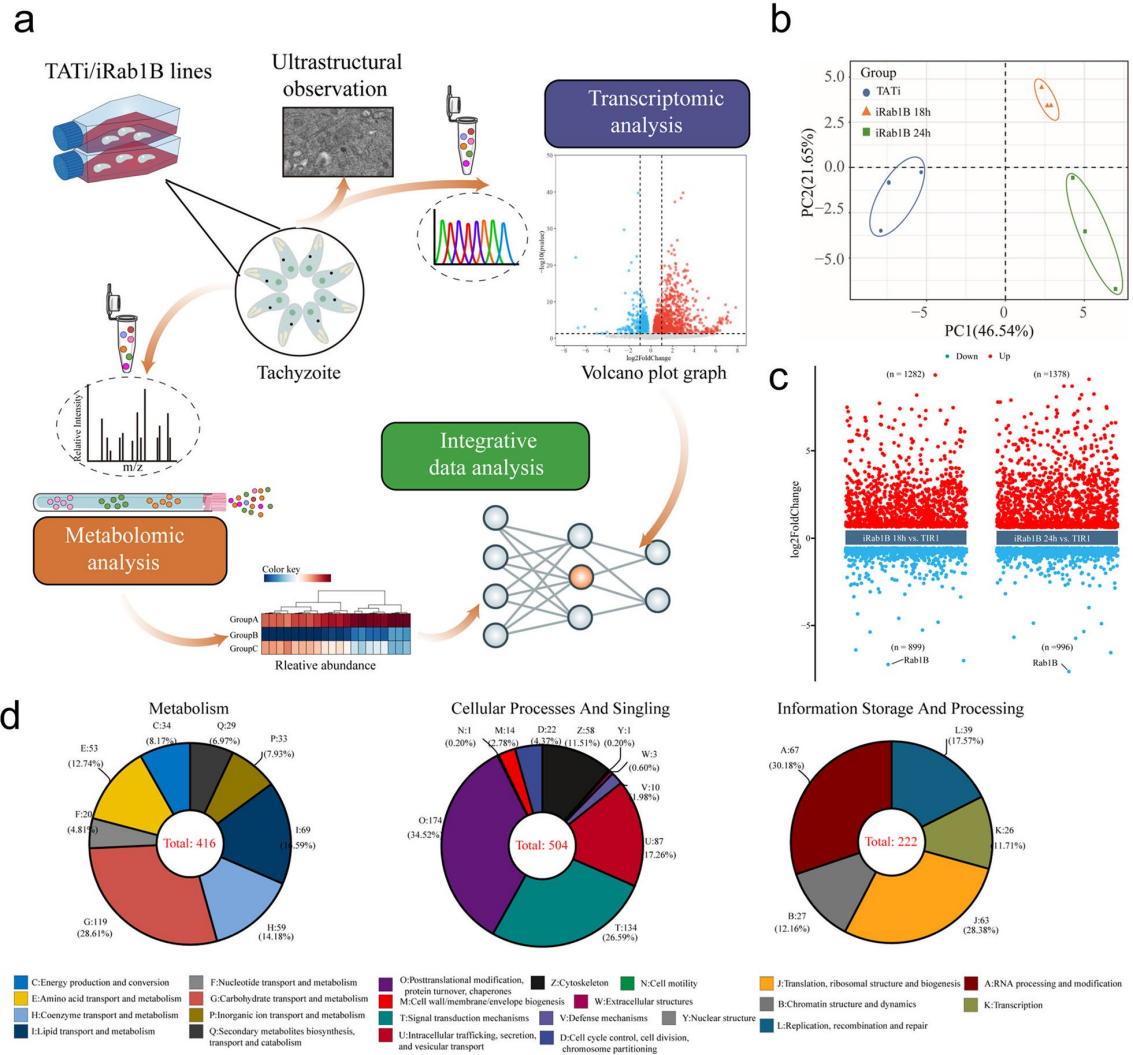
Experiment overview and preliminary presentation of the transcriptomic data. **a** Schematic illustration of the experimental design. Fresh tachyzoites were extracted and treated with ATc after Rab1B degradation for 0, 18 and 24 h. **b** Principal component analysis (PCA) plots for all RNA-Seq data. **c** Volcano plots showing DEGs after the degradation of TgRab1B treated with ATc for 18 h and 24 h; the reds dots and blue dots represent up- and downregulated genes, respectively. **d** COG analysis of identified DEGs from the above two comparisons. The number of DEG annotated genes involved in metabolism, cellular processes and signaling and information storage and processing in the EggNOG database

To understand the essential nature of Rab1B in the parasites, we designed a workflow for the TATi/Ty-Rab1B grown with or without inducer (18 and 24 h), where combined approaches of transcriptomics and metabolomics were utilized (Fig. 1a). First, we performed a data quality control for the transcriptomic datasets, which were calculated and listed in Additional file 2: Table S2. After high-throughput sequencing and quality control filtering of raw reads, approximately 78G clean reads with an average of 8.7G clean data per sample were generated, with average Q20 and Q30 ratios of 98.4% and 95.7%, respectively. After aligning clean data to the *T. gondii* reference genome, the mapping percentages of samples ranged from 93.94 to 95.88% for the parental line TATi, 85.74 to 94.13% for the iRab1B 18-h group and 70.01 to 84.88% for the iRab1B 24-h groups, respectively. The correlation heatmap revealed the consistency among the three biological replicates with high correlation in each individual cluster (Additional file 3: Fig. 2a). Principal component analysis (PCA) clearly separated the TATi group from the two treatment groups based on PC1, which contributed 46.54% variation and was the dominant component (Fig. 1b). In addition, the distribution of gene expression patterns was also scaled to a consistent level after FPRM normalization across all samples (Additional file 3: Fig. 2b). Meanwhile, all the gene expression levels in different groups were recorded (Additional file 4: Table S3). Together, the above results suggested good global indicators for sequencing accuracy.

**Fig. 2:**
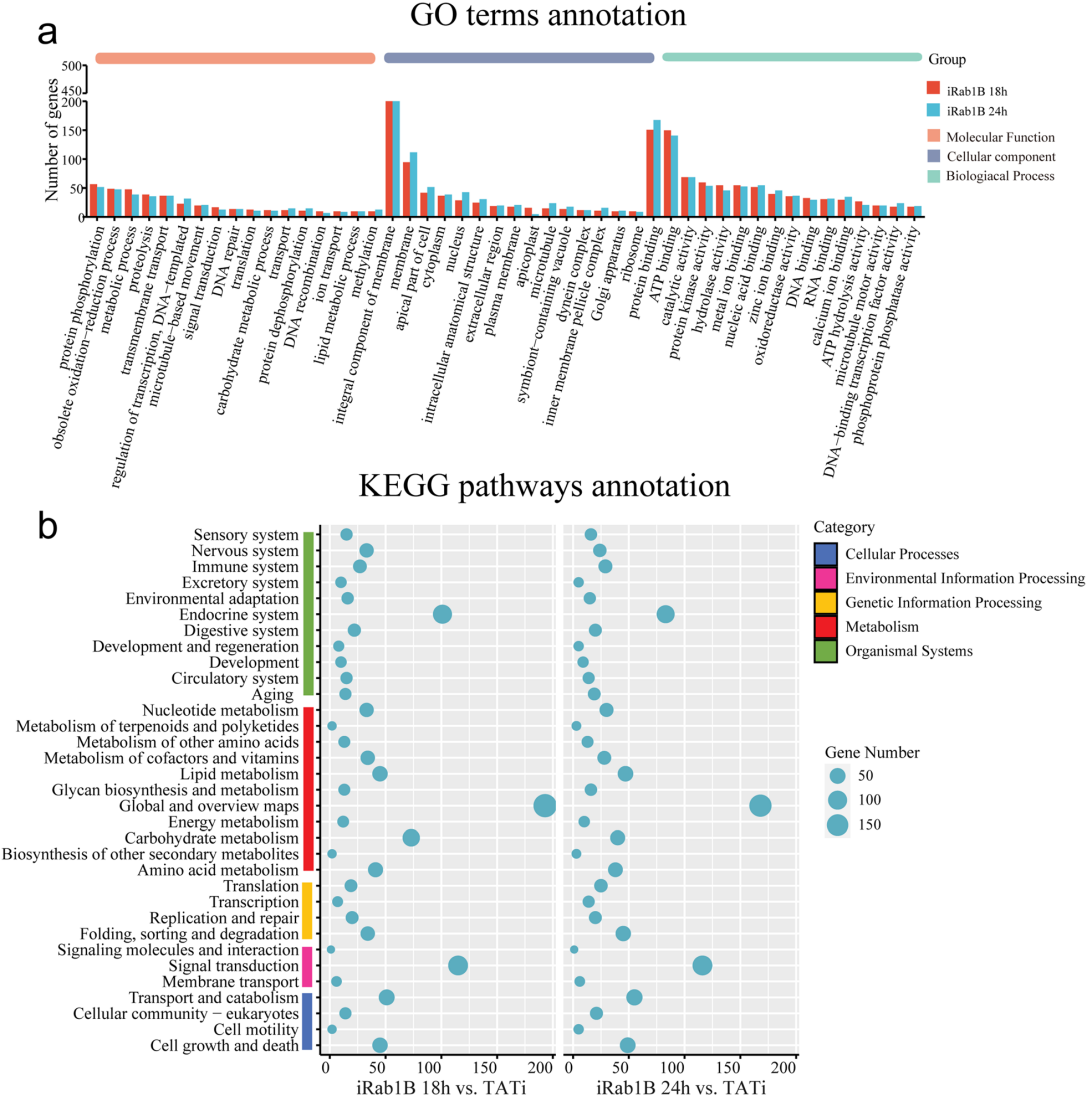
Functional classification and annotation of the DEGs from two comparisons in the GO and KEGG databases. **a** Bar plot of the GO functional annotation targeting the biological process (BP), cell component (CC) and molecular function (MF). **b** Bubble plot of KEGG classification targeting to the Cell Processes, Environment Information Processing, Genetic Information Processing, Metabolism and Organismal Systems categories. Size represents the number of genes, and color represents the *P* value

In the study, differential expression analysis yielded 2181 DEGs (1282 up- and 899 downregulated) and 2374 DEGs (1378 up- and 996 downregulated) for the 18-h iRab1B versus TATi and 24-h iRab1B versus TATi comparisons. Furthermore, volcano plots clearly presented the results of DEGs for the treatment group at 18 h and 24 h (Fig. 1c). Notably, the expression level of Rab1B gene displayed the greatest regulation, with a significant diminishment of Log_2_Foldchange of − 6.9 and − 7.3 in the two comparisons, respectively.

### Functional classification of DEGs identified in Rab1B-depleted parasites

According to the functional annotation for these DEGs using EGGNOG database, of a total of 3155 DEGs from the two comparisons, 416, 504 and 222 genes were mapped to known functional classifications pertaining to “metabolism,” “cellular processes” and “signaling and information storage and processing,” respectively (Fig. 1d). Specifically, nearly half of annotated genes were related to carbohydrate and lipid-related metabolism. These results indicated that Rab1B degradation caused a dramatic disruption in gene expression and stress response in the parasite. To understand the biological characteristics of the jointed DEGs from two comparisons, we first mapped these genes into GO database based on WEGO platform (Fig. 2a), which generated 48 subcategories from three main categories including biological process (BP), molecular function (MF) and cellular component (CC), respectively. The results showed that the top three BP terms were “protein phosphorylation,” “obsolete oxidation-reduction process” and “metabolic process.” The top three CC terms were “integral component of membrane,” “membrane” and “apical part of cell.” In the MF category, “protein binding,” “ATP binding” and “catalytic activity” were the most abundant items.

To gain further insights into metabolic pathways, we divided these DEGs into five categories via KEGG database (Fig. 2b), namely, “Cellular Process,” “Environmental Information Processing,” “Genetic Information Processing,” “Metabolism” and “Organismal Systems.” These categories were further divided into 33 subcategories, with the largest number of subclasses identified in the “Metabolism” and “Organismal Systems” categories. From top to bottom, the most abundant DEGs in both comparisons were associated with the “Endocrine system” subcategory within the “Organismal Systems” category. Additionally, of these 11 subcategories of “Metabolism” of both comparisons, “Global and overview maps” were enriched with most DEGs, followed by “Carbohydrate metabolism” and “Lipid metabolism,” which might be highly related to the selective nutrient salvage from host cell compartments or de novo biosynthesis for the parasite due to the depletion of TgRab1B.

### Shared DEGs in parasites induced at two different induction time points

**Fig. 3:**
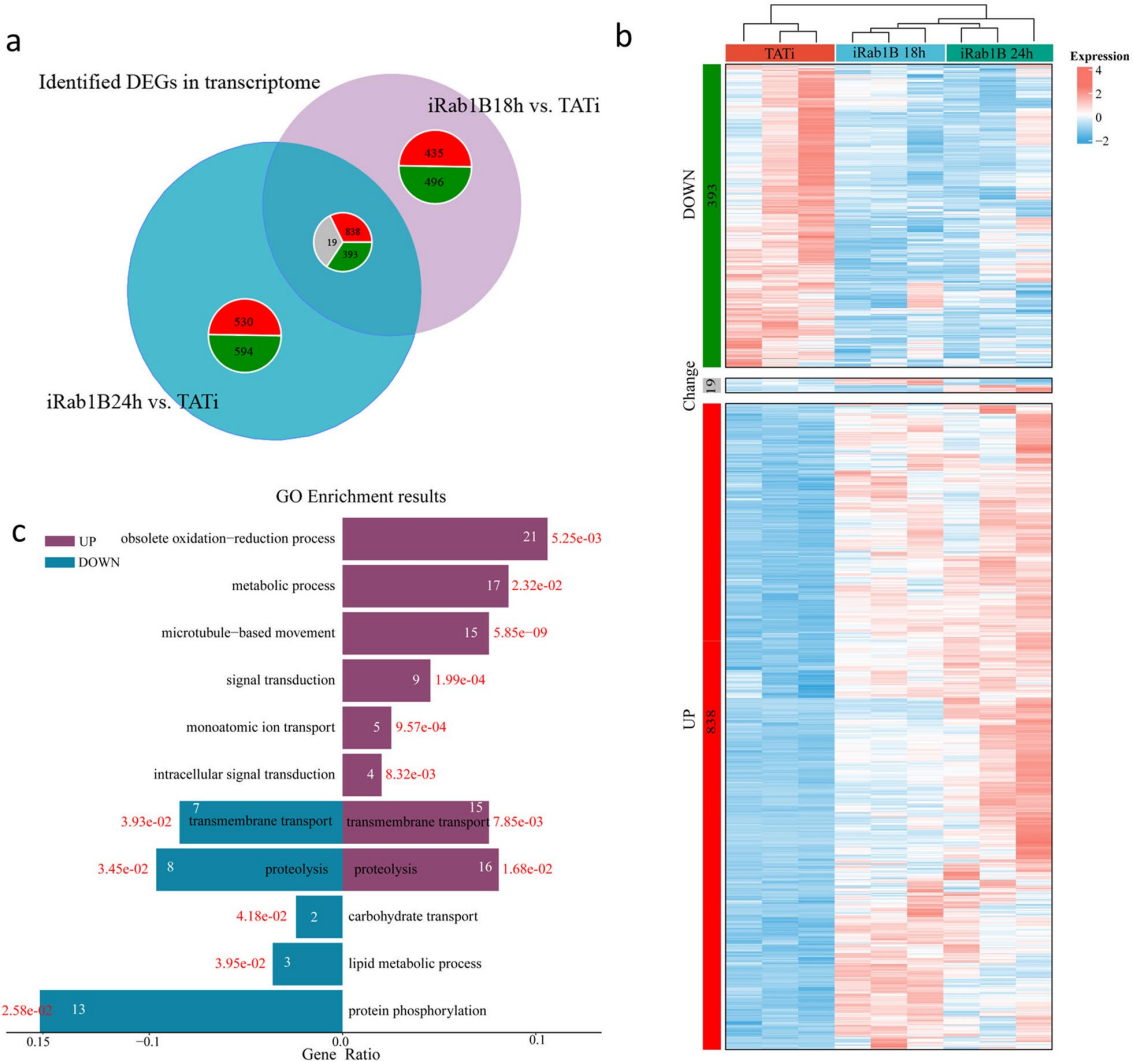
Common effect at the two different induction time points. **a** Venn diagram showing the differentially expressed genes at 18 h and 24 h after Rab1B degradation. **b** Hierarchical clustering heatmap exhibiting the expression level of shared genes among the three groups. The data are shown in three biological replicates. Expression value is normalized so that blue represent low expression and red represents high expression. **c** Bar plot showing gene ontology (GO) enrichment results of the shared DEGs. The *X*-axis labels indicate an enrichment ratio, and the *Y*-axis denotes the names of GO terms. The white and red numbers represent annotated gene numbers and significance, respectively

The common genes were further screened to evaluate the joint effects when subjected to the different time points. According to Venn analysis from the two comparisons, we observed 1250 shared genes, including 1231 genes with a consistent trend in regulation (838 up- and 393 downregulated) and 19 with an opposite trend, while 931 and 1124 DEGs were only significantly expressed in the iRab1B 18 h versus TATi and iRab1B 24 h versus TATi groups (Fig. 3a). Furthermore, the gene expression level changes and the detailed annotation for the 1250 DEGs were also displayed (Fig. 3b; Additional file 4: Table S3). By GO enrichment analysis, two GO terms, “transmembrane transport” and “proteolysis,” were simultaneously highly enriched by the up- and downregulated gene sets. Additionally, the downregulated genes were found to be mainly associated with the GO terms “protein phosphorylation,” “carbohydrate transport” and “lipid metabolic process” (Fig. 3c).

### Dynamic transcriptomic changes identified in Rab1B-depleted parasites

**Fig. 4:**
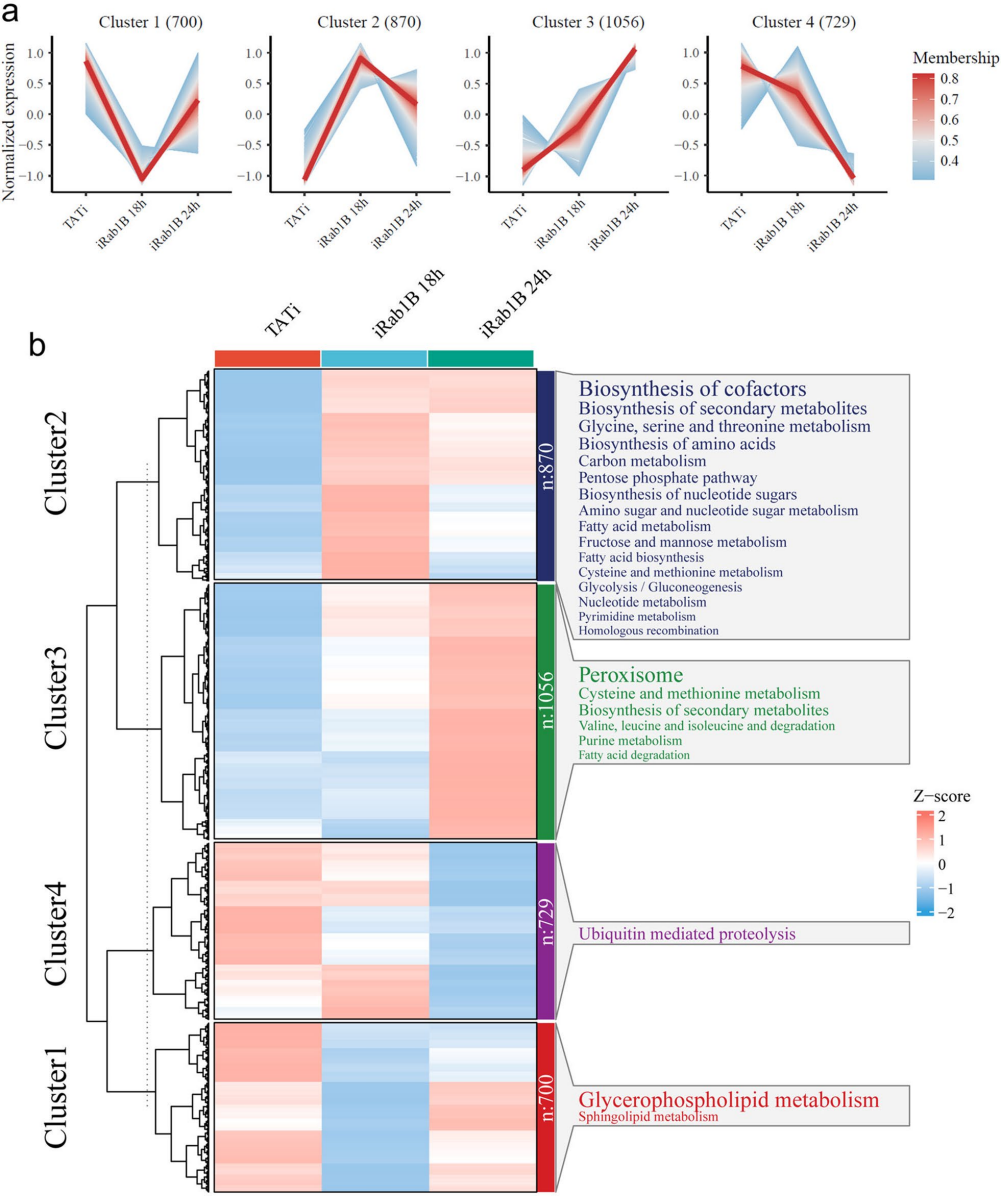
Characterizations of dynamic gene expression profiles of all DEGs. **a** Clustering line plot with different variation tendencies according to the fuzzy C-mean algorithm. **b** Heatmap of each expression pattern illustrates gene expression changes in different groups. Beside each gene cluster, the cluster size and representative enriched pathways are noted on the right-hand side. The size of terms in each box represents the corresponding significance

To better explore the dynamic changes in the gene expression of DEGs across the three groups in greater detail, we performed a clustering trend analysis using fuzzy c-mean algorithms. The expression levels of the 3155 DEGs were shown in a heatmap and were grouped into four expression patterns according to their variation tendency (Fig. 4a). Clusters 1, 2, 3 and 4 include 700, 870, 1056 and 729 genes, respectively. Compared to the TATi control, gene expression levels in clusters 1 and 4 showed a decreased trend, while the opposite was observed in clusters 2 and 3. As shown in Fig. 4b, significant enriched KEGG pathways (*P* < 0.05) were also displayed on the right-hand side of the heatmap. Compared to the control group, DEGs of clusters 2 and 3 enriched more pathways than those in clusters 1 and 4. These enriched pathways mainly included serval pathways associated with carbohydrate, amino acid and lipid metabolism. In particular, two additional lipid metabolisms, glycerophospholipid and sphingolipid metabolism, were highly enriched by DEGs in cluster 1. One gene encoding a PAP2 superfamily protein (TGGT1_247360), along with Rab1B and another phosphatase family protein (TGGT1_249030), was enriched together in sphingolipid metabolism. In addition to the three genes, a key sphingolipid synthase (TGGT1_246490) also showed lower expression levels in the treatment groups at iRab1B 18 h and 24 h compared with the control group.

**Fig. 5:**
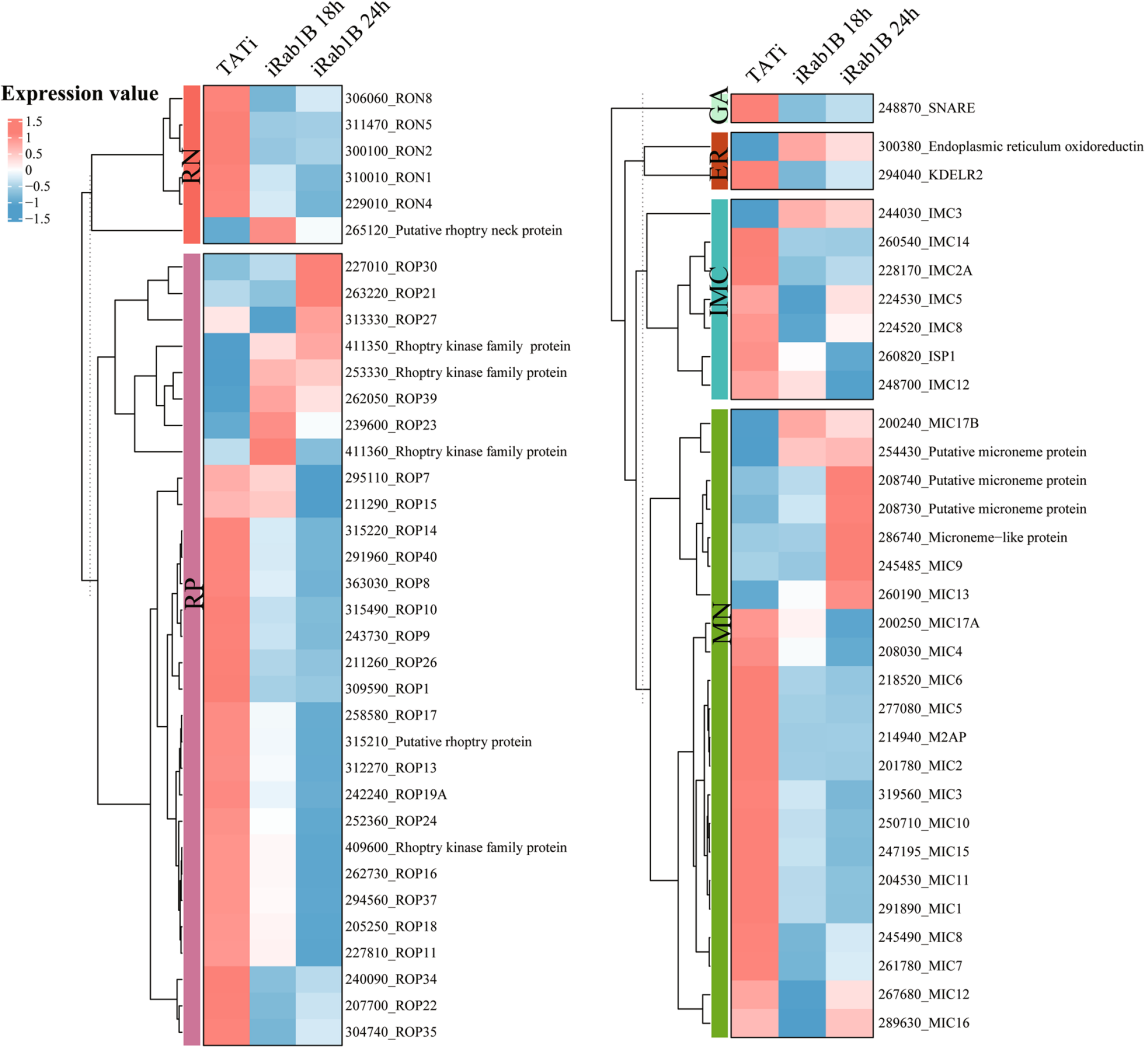
Heatmap shows the significantly altered genes involved in the different organelles of *Toxoplasma gondii*. The log2 treated/control fold change values of transcripts were normalized and scaled by z-score in each. Colors represent the gene expression level of each DEG, from blue (low) to red (high). RN: rhoptry neck; RB: rhoptry bulb; GA: Golgi apparatus; ER: endoplasmic reticulum; IMC: inner membrane complex; MN: microneme

Given the previous evidence that Rab1B impaired the biogenesis of rhoptries, it is necessary to ascertain the expression level changes of organelle proteins due to Rab1B degradation; therefore, we mainly focused on these DEGs localized to rhoptry (RP), microneme (ME), inner membrane complex (IMC), Golgi apparatus (GA) and endoplasmic reticulum (ER) (Fig. 5). Intriguingly, among these DEGs, a subset of rhoptry proteins (ROPs and RONs) was found to be significantly downregulated upon Rab1B degradation. Moreover, most of DEGs in the other four organelles caused varying degrees of decline in expression level. Detailed information on gene expression levels and fold changes in each cluster are presented (Additional file 5: Table S4).

### Validation of examples of DEGs by qRT-PCR

**Fig. 6:**
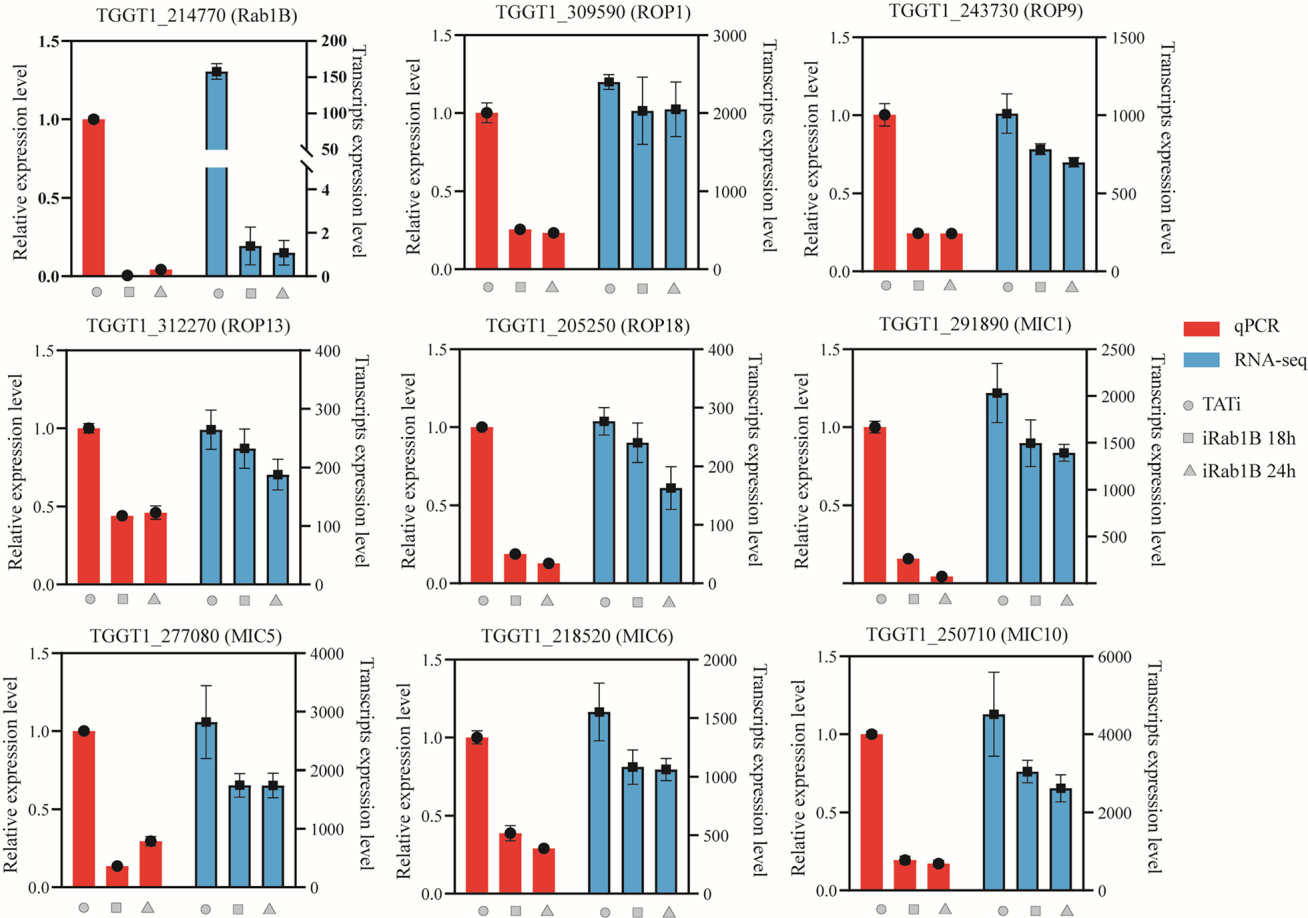
Verification of the expression patterns of RNA-seq results by qRT-PCR. These candidate genes included Rab1B, four rhoptry proteins (ROP1, ROP19, ROP13 and ROP18) and four microneme proteins (MIC1, MIC5, MIC6 and MIC10). The bars represent the standard deviations (± SD) of the mean

To verify the reliability of the RNA-seq data in our study, we selected Rab1B and eight organelle proteins and examined their expression at three different time points by qRT-PCR. The expression patterns of all detected gene mRNA levels were consistent with the qRT-PCR results, showing that the RNA-seq data were accurate and reliable (Fig. 6).

### Metabolics verifies metabolic roles of Rab1B

**Fig. 7:**
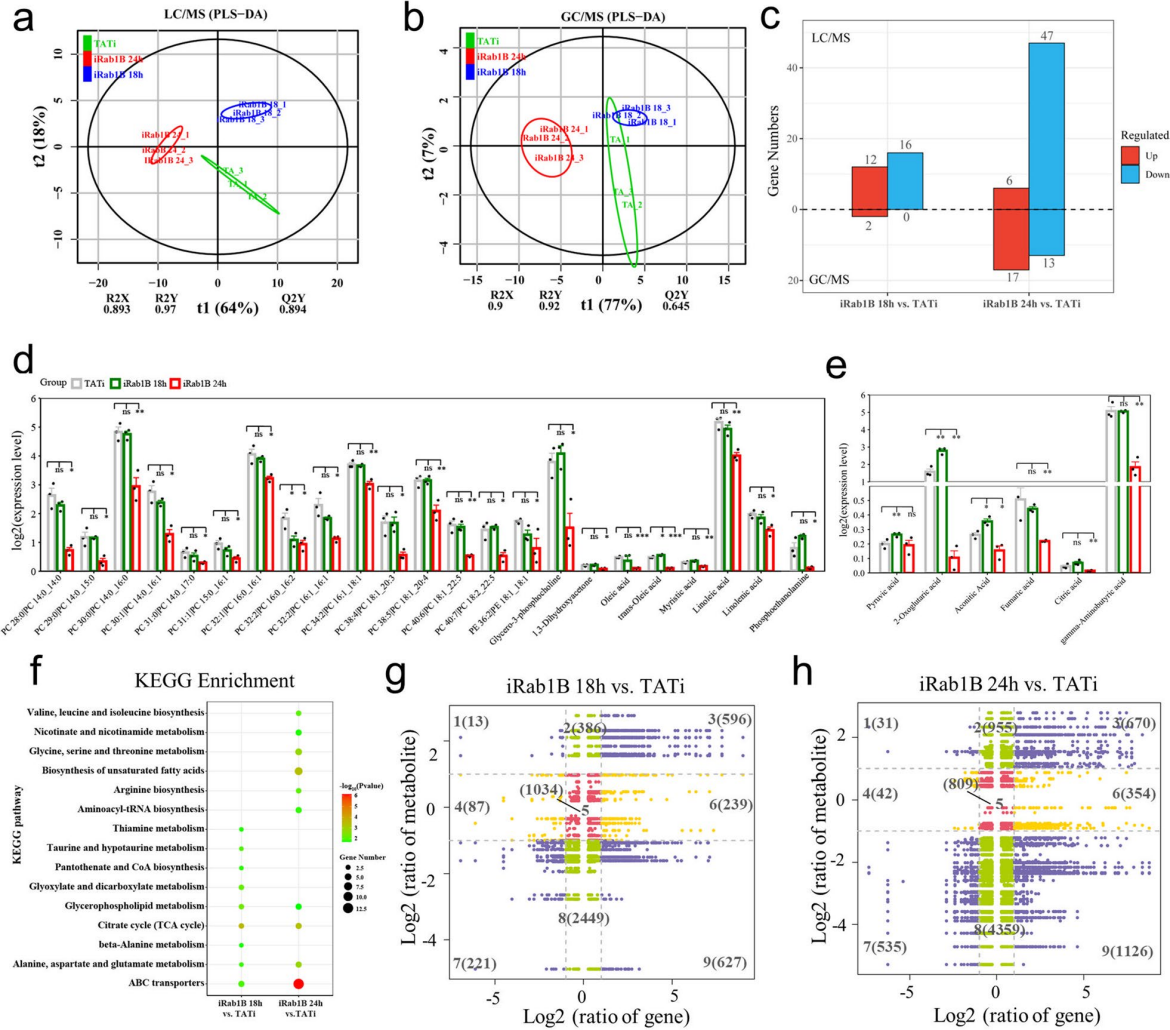
Overview of the metabolic data from the different comparison groups. PLS-DA of comparisons LC-MS/MS **a** and GC-MS/MS **b** analysis of the obtained metabolic profiles. **c** Quantitative statistics of identified differential metabolites by LC-MS/MS and GC-MS/MS platforms. Comparative bar plot shows the identified metabolites included fatty acids and lipids **d**, such as myristic acid, oleic acid and phosphatidylcholine (PC) and metabolites related to TCA cycle (**e**) (mean ± SEM; *n* = 3; *P < 0.05; **P < 0.001; ***P < 0.0001). The legend for the two plots is the same and shown at the upper left corner. Metabolic data of two methods are available in the Excel sheets (Additional file 6: Table S5). **f** KEGG enrichment bubble diagram for the identified metabolites from the LC-MS/MS and GC-MS/MS methods. Correlation analysis of metabolomic and transcriptomic data for the iRab1B 18 h versus TATi (**g**) and iRab1B 24 h versus TATi (**h**) comparisons; the nine quadrant diagrams indicated the correlation of compounds (identified by metabolomic analysis) and genes (obtained from RNA-seq). Purple, green, yellow and red points show DEG and DEM pairs, non-DEG and DEM pairs, DEG and non-DEM pairs, and non-DEG and non-DEM pairs, respectively

To better study the differences in metabolite compositions in different comparative groups, LC-MS/MS and GC-MS/MS analyses were utilized together to detect alterations in metabolite compositions. In total, 69 differential metabolites in LC-MS/MS analysis (35 in positive mode and 34 in negative mode) and 30 differential metabolites in GC-MS/MS analysis were detected and used for downstream processing (Additional file 6: Table S5). Through multivariate analysis using PLS-DA, we were able to preliminarily understand the overall metabolic differences among the three groups. As shown in Fig. 7a, b, the results showed clear distinctions among the three groups. These nice indicators, including R2X and R2Y values close to 1 and suitable Q2 values, demonstrated the precision and repeatability of the two methods used in the study.

In the LC-MS/MS analysis, compared with the metabolites in the TATi group, 28 metabolites were significantly altered at 18 h in the early-stage comparison, of which 12 were upregulated and 16 were downregulated. At 24 h in the late-stage comparison, 53 metabolites were significantly changed, with 6 upregulated and 47 downregulated. However, fewer DEMs were identified in GC-MS/MS analysis than in the LC-MS/MS. Only two significantly upregulated metabolites were found in the early comparison, while a stronger influence occurred in the late comparison, as revealed by the 17 upregulated and 13 downregulated metabolites (Fig. 7c). Notably, these altered DAMs include several phosphatidylcholines (PCs), fatty acids and lipids such as (trans)-oleic acid (C18 fatty acid), myristic acid (C14 fatty acid) as well as other metabolites related to the TCA cycle (Fig. 7d, e), all of which decrease more at the 24 h time point after Rab1B degradation. Afterward, we performed KEGG enrichment analysis on the DAMs identified by the LC-MS/MS and GC-MS/MS approaches to identify the main metabolic pathway. (Fig. 7f). All differential metabolites were co-enriched in 15 biological pathways (*P* < 0.05). Expectedly, several vital pathways were potentially related to lipid metabolism-related and carbohydrate metabolism pathways in both comparisons, such as the citrate cycle (TCA cycle), glycerophospholipid metabolism and biosynthesis of unsaturated fatty acids.

### Combined analysis of the transcriptome and metabolome

**Fig. 8:**
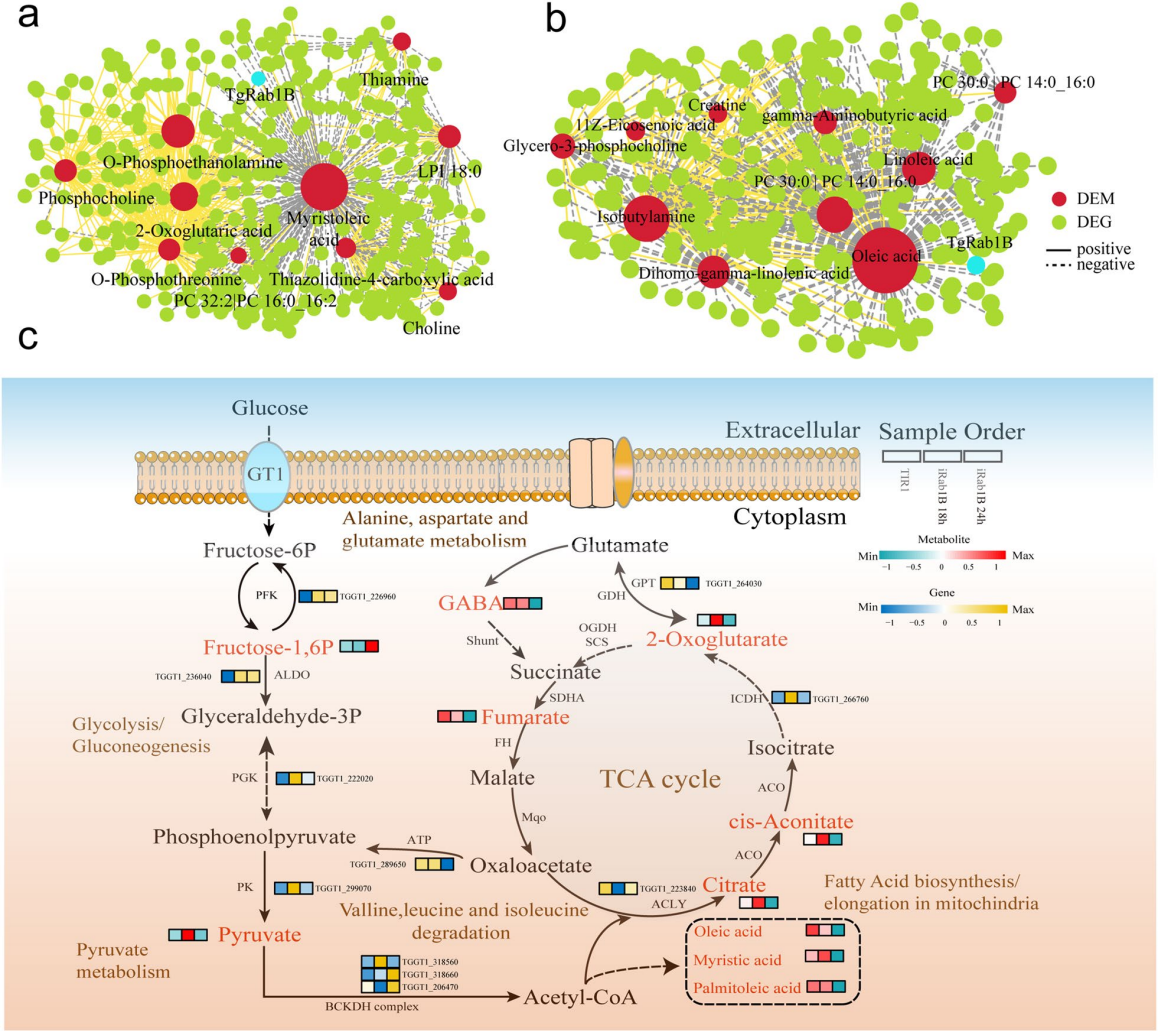
Integration analysis of metabolomic and transcriptomic data. Interaction networks between top 10 DEMs correlated with screened DEGs (|log2FoldChange > 1 and *P* < 0.05) in **a** iRab1B18h versus TATi and **b** iRab1B 24H versus TATi comparisons were visualized with Cytoscape, wherein the yellow solid lines indicate the positive correlations with *R* > 0.95 and *P* < 0.05, while the gray dashed lines indicate the negative correlations with *R* < 0.95 and *P* < 0.05). **c** Map of expression profiles of DEGs and DEMs associated with central carbon pathways in *T. gondii*. The heatmap represents the corresponding expression level of the involved DEGs and DEMs with the sample order at the right upper corner, wherein the node colors ranging from blue to yellow indicate the number of metabolites and the node colors ranging from green to red indicate the number of metabolites

To further investigate the integrative biological information from multi-omics data, variations of all DEMs and DEGs in each comparison were shown in nine-quadrant diagrams with |*R*| > 0.95 and *P* < 0.01 (Fig. 7g, h). Briefly, the DEMs and DEGs presented in quadrants 3 and 7 are positively correlated, while those in quadrants 1 and 9 were negatively correlated. To investigate the metabolites with the most changes, correlation network diagrams were used to examine the connections between the top 10 changed DEMs and major DEGs in the two comparisons (Fig. 8a, b). Among these displayed DEMs, two fatty acid metabolites, myristoleic acid and oleic acid, had the most genes and exhibited strong negative correlation (*R* < − 0.95, *P* < 0.05) with Rab1B.

Furthermore, we proposed a model for the alteration of transcriptomic and metabolic level in response to central carbon metabolic pathways after Rab1B degradation. Here, only the pathways containing DEGs and corresponding DEMs were presented. For the TCA cycle, major metabolites such as citric acid, cis-aconitate and 2-oxoglurate were remarkably reduced during the late stage of ATc induction (Fig. 8c) in contrast to their expression changes at 18 h. Likewise, the expression of several lipids, including oleic acid, myristic acid and palmitoleic acid, showed a similar trend, which may contribute to the significant accumulation of fatty acid biosynthesis and elongation. Correlation analysis suggests that these DEGs might play a direct or indirect regulatory role in key DEM metabolism throughout the whole developmental period. Collectively, these results could help us to comprehensively understand the molecular basis during the stage and further support the essential role of Rab1B.

## Discussion

*Toxoplasma gondii* is a powerful system for studying parasite molecular biology because of its ease of culture, haploid genome and genetic tractability. Our recent study demonstrated that the parasite relies on the micropore to initiate and undertake the endocytic process for salvage of nutrients, such as host cytosolic materials (e.g. proteins and biotin) and Golgi ceramide [21]. This suggested that the parasite endocytic process contributes to the fatty acid biosynthesis in the apicoplast and lipid metabolism in the parasites, which was further supported by the transcriptomic and metabolic analysis, while how the mitochondrial metabolism is affected by the micropore remains unknown. The endocytic process clearly contributes to the nutrient status and parasite metabolism in the apicoplast and mitochondrion. Our further work clarified the key regulators involved in endocytic trafficking, and the regulators included the key GTPase Rab1B [23]. However, the essential nature of Rab1B needs to be further explored in the parasites. Here, we provided further evidence from an integrated multi-omics analysis. Differential expression analyses identified thousands of DEGs upon depletion of the key regulator Rab1B. Furthermore, combined with the metabolome data, some candidate DEGs are found to be mainly related to biosynthesis of fatty acids and lipids and the TCA cycle, indicating that altered expression of Rab1B has a more significant impact on specific nutrient salvage of *T. gondii*.

In eukaryotic cells, many steps of membrane trafficking within the secretory and endocytic pathways, including the formation, scission, transport, tethering and eventual fusion of membrane-bound organelles and vesicles, are thought to be regulated by small Rab GTPases [25–27]. Rab1B generally contributes to upstream membrane traffic, namely, ER-Golgi, and it is required for the initiation of secretion and autophagy [28, 29]. In *T. gondii*, Rab1B was found to be involved in the protein sorting to the rhoptry bulb at the early induction time points in the TATi line. Our integrated analyses suggest that depletion of Rab1B is associated with metabolic defects and resulted in gene expression changes related to secretory organelles including the rhoptries and micronemes. The detailed omics results supported our previous findings and furthered our understanding of Rab1B on the endocytic and protein trafficking.

In our transcriptomic data, EggNOG and KEGG databases together indicted that the identified DEGs from two comparisons were mainly involved in the carbohydrate and lipid-related metabolism, providing valuable clues for our further metabolic analysis. It is well known that the tachyzoite membranes contain several phospholipids, such as sphingomyelin (SM), phosphatidyl threonine (PtdEtn), phosphatidylcholine (PtdCho) and phosphatidylserine (PtdSer) [30, 31]. As outlined above, glycerophospholipid and sphingolipid metabolisms were found to be enriched by the downregulated genes from cluster 1 compared to control parasite lines. Although sphingolipid metabolism was significantly enriched by only three DEGs, some genes in the same cluster may play a similar role, as indicated by the expression change of the key SM synthase (TGGT1_246490). In addition, the long fatty acid belonging to sphingolipid, ceramide, has been reported to act as a secondary messenger in ubiquitous organisms [32]. The ubiquitin-meditated proteolytic pathway shown in this study was significantly enriched by the genes of cluster 2, indicating Rab1B degradation indeed played an important role in the scavenging of sphingolipids. Beyond these lipid-related pathways, additional pathways in these enrichment results also deserve to be investigated in future studies.

Recent reports revealed that *T. gondii* scavenges various fatty acids and lipids from the host cells, including phospholipids and cholesterol, some of which are further metabolized by the parasite [9, 10, 33]. Another study also indicated that most Rab family proteins carried lipid modifications that were necessary for membrane recruitment [34]. In more detail, we directly observed that most metabolites related to (unsaturated) fatty acids and lipids all exhibited the lowest expression level in the late stage at 24 h (Fig. 7d, e), indicating a very strong accumulation of growth defects in parasite replication during the induction time window of 18–24 h. Among these altered metabolites, phosphoethanolamine was known to be an ethanolamine derivative used to construct two different categories of phospholipids [35]. One category is termed a glycerophospholipid and the other a sphingomyelin, or more specifically, within the sphingomyelin class, a sphingophospholipid. These results also reveal our omics data at the transcriptomic and metabolic level are partially consistent and reliable. Notably, in addition to exogenous acquisition, the parasite has can synthesize de novo [36]. However, the ability of the parasite to scavenge phospholipid from the host may be complicated, and clearly the balance between de novo synthesis and scavenging merits further investigation.

In the study, we shed light on the gene expression of and metabolic responses to the degradation of Rab1B protein in *T. gondii*, along with some evidence from TEM observations. Characterization of the differentially expressed genes and metabolites contributes to a broader understanding of the secretary pathway and sophisticated pathogenicity under specific conditions. Our findings further examined the essential role of Rab1B and suggested that the working mechanism of Rab1B in *T. gondii* may be reshaped during evolution, expanding our understanding of the function and evolution of small GTPase in the parasite and other protists.

## Conclusions

Given the essential role of Rab1B in vesicular transport, multi-omics and TEM analyses were investigated together to determine changes in the parasite upon depletion of Rab1B. Our findings revealed that depletion of Rab1B led to disorganization of the endo-membranous system and had an overall effect on genes involved in metabolism and organelle biogenesis, suggesting Rab1B is a key regulator functioning in the nutrient salvage process and organelle formation in the parasite.

## Materials and methods

### Parasite and cell culture

The parental line RHΔku80Δhxgprt/TATi and its derived anhydrotetracycline (ATc)-inducible parasite line TATi/Ty-Rab1B (here called TATi/iRab1B) based on tetracycline transactivator-based inducible system (TATi) used in the study were constructed and stored in our laboratory. The constructed strategy and detailed materials including corresponding primers and plasmids are provided in our recently published paper [23]. These parasite lines were grown on human foreskin fibroblast (HFF) cells (ATCC, SCRC-1041) in DMEM (D5 medium) supplemented with 5% heat-inactivated fetal bovine serum, 2 mM glutamine and 100 units penicillin-streptomycin at 37 °C with 5% CO_2_. The TATi/iRab1B parasites were grown in HFF cells and induced by ATc for 18 and 24 h, while parental parasites were induced at ATc for 0 h to serve as the control. Parasites were harvested in chilled PBS and immediately quenched at – 20 °C for RNA-seq and metabolomics analyses.

### Electron microscopic observation

Parasites grown on HFF cell monolayers were collected by scraping them off and fixing them in a fixative mixture of 2% paraformaldehyde and 3% glutaraldehyde at 4 ℃ overnight. The samples were washed, fixed in 1% osmium tetroxide in 50 mM phosphate buffer at 4 ℃ for 1 h, washed three times and dehydrated with sequential concentrations of ethanol and of acetone and Spurr for 1 h. The samples were then imbedded in Spurr, followed by sectioning to 100-nm slices with a Leica ultramicrotome (EM UC7). The ultrathin sections were collected on 100-mesh grids. The grids were visualized by JEOL transmission election microscopy (JEM1400FLASH).

### RNA extraction and illumina sequencing

TATi/iRab1B parasites were grown in the presence of ATc for 0, 18 and 24 h. The fresh tachyzoites (~ 1 × 10^7^) from the three time points were harvested by passing through 22G needles and filtration through 3.0-micron polycarbonate membranes after washing with cold PBS. Three groups of parasites with three biological replicates were established. Total RNA was extracted from the samples using TRIzol Reagent kit (Invitrogen, Carlsbad, CA, USA) according to the manufacturer's instructions. RNA quality was determined by an Agilent 2100 Bioanalyzer (Agilent Technologies, Palo Alto, CA, USA) and checked using RNase free agarose gel electrophoresis. High-quality RNA samples with OD260/280 ranging from 1.8 to 2.2 and RIN ≥ 6.5 were used for sequencing cDNA libraries following the TruSeqTM RNA sample preparation kit from Illumina (San Diego, CA, USA). Messenger RNA was isolated according to poly A selection method by oligo(dT) beads and then fragmented by fragmentation buffer. Then, double-stranded cDNA was synthesized using a SuperScript double-stranded cDNA synthesis kit (Invitrogen, Carlsbad, CA, USA) with random hexamer primers (Illumina). Subsequently, the synthesized cDNA underwent end repair, phosphorylation and “A” base addition according to Illumina’s library construction protocol [37]. Libraries were size selected for cDNA target fragments of 300 bp on 2% Low Range Ultra Agarose and PCR amplified using Phusion DNA polymerase (NEB) for 15 cycles. After quantification by TBS380, RNA-seq sequencing library was sequenced on an Illumina NovaSeq 6000 sequencer by Gene Denovo Biotechnology Co. with a paired-end 150-bp strategy (Guangzhou, China).

### Transcriptomic data processing

To ensure high-quality data for analysis, raw sequencing reads were first processed to remove adapters or low-quality bases using fastp software (version 0.18.0) [38]. Reads containing > 10% unknown nucleotides (N) and > 50% bases with a Q-value ≤ 20 bases were filtered out. After that, the remaining high-quality data of each sample were separately aligned to the *T. gondii* reference genome, downloaded from the ToxoDB database (https://toxodb.org/, release 63), using Hisat2 with default parameters (version 2.1.0). The mapped reads were assembled by using a reference-based approach with StringTie (version 2.2.1) [39]. The gene expression counts were quantified via RSEM software (version 1.3.1) [40] and then transformed using the FPKM (fragment per kilobase of transcript per million mapped reads) method. Principal component analysis (PCA) and correlation analyses were performed to evaluate the sample similarities based on the normalized gene expression level.

The DESeq2 (v1.30.0) package [41] was utilized to identify the differentially expressed genes (DEGs) between two comparison groups (iRab1B 18 h vs. TATi and iRab1B 24 h vs. TATi). Genes with a *P* value < 0.05 and an absolute fold change ≥ 1.5 were considered as DEGs. We combined all DEGs from two comparisons to perform Fuzzy C-means clustering trend analysis using the “Mfuzz” package [42]. Additionally, “ClusterProfiler” package (v3.18.0) [43] was used to conduct the Gene Ontology (GO) and Kyoto Encyclopedia of Genes and Genomes (KEGG) enrichment analysis targeting the different gene sets in our downstream analysis, with a significant cut-off level of *P* value < 0.05.

### Quantitative real-time PCR (qRT-PCR) analysis

The enriched mRNA with 100 to 500 ng for each sample was reverse transcribed into cDNA according to HiScript III 1st Strand cDNA Synthesis Kit (+ gDNA wiper) (Vazyme, Nanjing, China) and random hexamers. The qRT-PCR analysis was carried out by the PerfectStart Green qPCR SuperMix (SYBR green) (TransGen Biotech, cat. no. AQ601) on a StepOneTM Real-Time PCR System (version 2.3). The reaction volume was 20 ul, and the procedure involved an initial step at 94 ℃ for 30 s, followed by 40 cycles of 94 ℃ for 5 s, 60 ℃ for 15 s and 72 ℃ for 10 s. *Toxoplasma gondii* actin protein (ACT1, TGGT1, 209030) was used as a reference gene to normalize gene expression, and the corresponding primers used were also recorded (Additional file 7: Table S1). The relative expression level of each candidate gene was calculated using the 2^−ΔΔCt^ method and visualized with GraphPad Prism 8. Quantitative data are presented as mean ± standard error of the mean.

### Untargeted metabolomics analysis by LC-MS/MS

Sample preparation procedures for untargeted metabolome were strictly carried out as previously described [21], and subsequent extraction, identification and quantification of metabolites were performed by ProfLeader Biotech Co., Ltd. (Shanghai, China). In brief, the instrument platform used for LC-MS analysis was the Thermo Fisher Ultimate 3000 UHPLC system equipped with an ACQUITY UPLC BEH C18 column (2.1 mm × 100 mm, 1.7 μm, Waters) maintained at 40 ℃, collecting data in standard positive and negative ion modes to optimize metabolite coverage. To equilibrate the systems, for the positive mode: the mobile phases contained water (solvent A) and methanol (solvent B), both with 0.1% formic acid. The following increasing linear gradient of solvent B (v/v) was used: 0 min, 2%B; 12 min, 95%B; 15 min, 100%B and held to 18.1 min; 18.1 min, 2%B and held to 20 min. For the negative mode: the mobile phases consisted of (A) water and (B) methanol/water (95:5, v/v), both with 6.5 mM ammonium bicarbonate. The linear gradient elution of solvent B was changed according to the following time scale: 0 min, 2%B; 9 min, 70%B; 14 min, 100%B and held to 18 min; 18.1 min, 2% B and held to 20 min. The flow rate was set to 0.25 ml/min, and the sample injection volume was 2 μl and 3 μl for the positive and negative mode, respectively.

The ESI-MSn experiments were performed on a Thermo Fisher Q Exactive Hybrid Quadrupole-Orbitrap Mass Spectrometry (QE) in Heated Electrospray lonization Positive (HESI +) and Negative (HESI-) mode as previously described. The obtained raw data were transformed to mzXML format by ProteoWizard and then imported into the R software platform for data processing using XCMS and CAMERA packages [44], including peak extraction and alignment and compound identification. Using the *m/z* (MSI), mass spectra (MS2) and retention times (RT) of compounds; metabolite identification was conducted against inhouse-developed databases, such as KEGG, Human Metabolome Database (HMDB) and Lipidmaps Databases. Data preprocessing, statistical analysis and metabolite and functional annotation were done based on the total integrated and normalized peak intensities using META-X software [45]. Furthermore, metabolic alterations among the different comparison groups were exhibited via partial least-squares discriminant analysis (PLS-DA) based on Pareto variance (Par) scaling using “ropls” package (v1.6.2). The identified metabolites were screened with *P* < 0.05 by Student’s *t*-test, and metabolites with variable importance in projection (VIP) score > 1 and absolute fold change > 1.5 were regarded as significant differential metabolites (DEMs). Enrichment of metabolic pathways using hypergeometric test was analyzed using MetaboAnalyst 5.0 server (https://www.metaboanalyst.ca/) [46] based on the sets with KEGG sets, in which *P* < 0.05 was considered significant.

### Untargeted metabolomics analysis by GC-MS/MS

The above parasite resuspension was evaporated with l-corleucine and dried under nitrogen stream. The resulting residue was dissolved in methoxyamine hydrochloride in pyridine and incubated at 37 °C for 90 min. Afterward, BSTFA (with 1% TMCS) was supplemented in the mixture and derivatized at 70 °C for 60 min prior to GC-MS metabolomics analysis. Quality control samples were pooled from all samples and processed using a consistent method. Instrumental analysis was performed on an Agilent7890A/5975C GC-MS system with an OPTIMA 5 MS Accent fused-silica capillary column (30 m × 0.25 mm × 0.25 μm, German). Helium (> 99.9%) was used as the carrier gas with 1 ml/min flow rate through the column. The injection volume was 1 μl, and the solvent delay time was 5 min. The initial oven temperature was increased from 60 °C to 320 °C and finally held for 4 min. The temperatures of injector, transfer line and electron impact ion source were set to 250 °C, 260 °C and 230 °C, respectively. Data were collected in full scan mode (*m/z* 50–600).

The peak picking, alignment, deconvolution and further processing of the raw data were conducted according to the previously published protocols [47]. The acquired data were exported as a peak table file, including observations (sample name), variables (rt_mz) and peak areas. Normalization was carried out against the total peak value of all peaks before downstream statistical analysis. The identification of differential metabolites and functional enrichment was performed using the consistent pipeline as described for the LC-MS analysis.

### Joint analysis of transcriptome and metabolome

A nine-quadrat graph was constructed to illustrate the fold change of DEMs and DEGs in each comparative group with a |*R*| > 0.95 and *P* < 0.01 threshold, and the Pearson correlation coefficients were assessed via pairwise comparison using the “Hmisc” package in R. To further observe the changes and associations of metabolites and genes, the top 10 DEMs and DEGs in each of the two comparisons were analyzed to show the correlation network diagrams using Cytoscape (v3.8.5).

### Supplementary Information

**Additional file 1.** Depletion of Rab1B caused disorganized endomembrane in parasites. The iRab1B parasites were grown in ATc for 18 and 24 h, followed by fixation, embedding and slicing for transmission electron microscopy (TEM). Normal endomembranes were observed in the TATi line (a), while defects of the endomembrane were observed in the iRab1B induced by ATc for 18h (b). The parasites with normal or abnormal endomembranes were quantified in the TATi and iRab1B lines when Rab1b was treated with ATc for 0, 18 and 24 h, respectively (c). Scale bar = 200 nm.

**Additional file 2.** Summary of RNA-sequencing results obtained in the study by Illumina NovaSeq 6000 platform.

**Additional file 3.** Global view of transcriptomic expression profiles during TgRab1B degradation. (a) Correlation heatmap analysis showing the relationship in the samples of the control and other two parasite lines. (b) Boxplot showing the FPKM-normalized expression distribution in different groups.

**Additional file 4.** Annotated results of all predicated coding genes and corresponding expression level in all samples.

**Additional file 5.** Fuzzy c-mean clustering results according to the Mfuzz package in R.

**Additional file 6.** Differential metabolites from iRab1B 18h vs. TATi and iRab1B 24h vs. TATi comparisons based on the LC-MS/MS and GC-MS/MS methods.

**Additional file 7.** Primers used for RT-qPCR analysis.

## Data Availability

The raw transcriptomic data and metabolic data reported in this paper have been deposited in the Genome Sequence Archive (GSA, https://ngdc.cncb.ac.cn/gsa) and OMIX (https://ngdc.cncb.ac.cn/omix), China National Center for Bioinformation/Beijing Institute of Genomes, China Academy of Sciences with accession number CRA008662 and OMIX002062, respectively.

## Abbreviations

DEGs: Differentially expressed genes
DEMs: Differentially expressed metabolites
ATc: Anhydrotetracycline
FPKM: Fragment per kilobase of transcript per million mapped reads
PCA: Principal component analysis
PLS-DA: Partial least-squares discriminant analysis
HFF: Human foreskin fibroblast
RT: Retention times
HMDB: Human Metabolome Database
GO: Gene Ontology
BP: Biological process
CC: Cellular component
MF: Molecular function
KEGG: Kyoto Encyclopedia of Genes and Genomes
