# Supplementary figures and images for: Transcriptomic and metabolomic analyses reveal the essential nature of Rab1B in Toxoplasma gondii
Source: Parasit Vectors. 2023 Nov 8;16:409. doi: 10.1186/s13071-023-06030-6 (PMC10634116; doi:10.1186/s13071-023-06030-6)

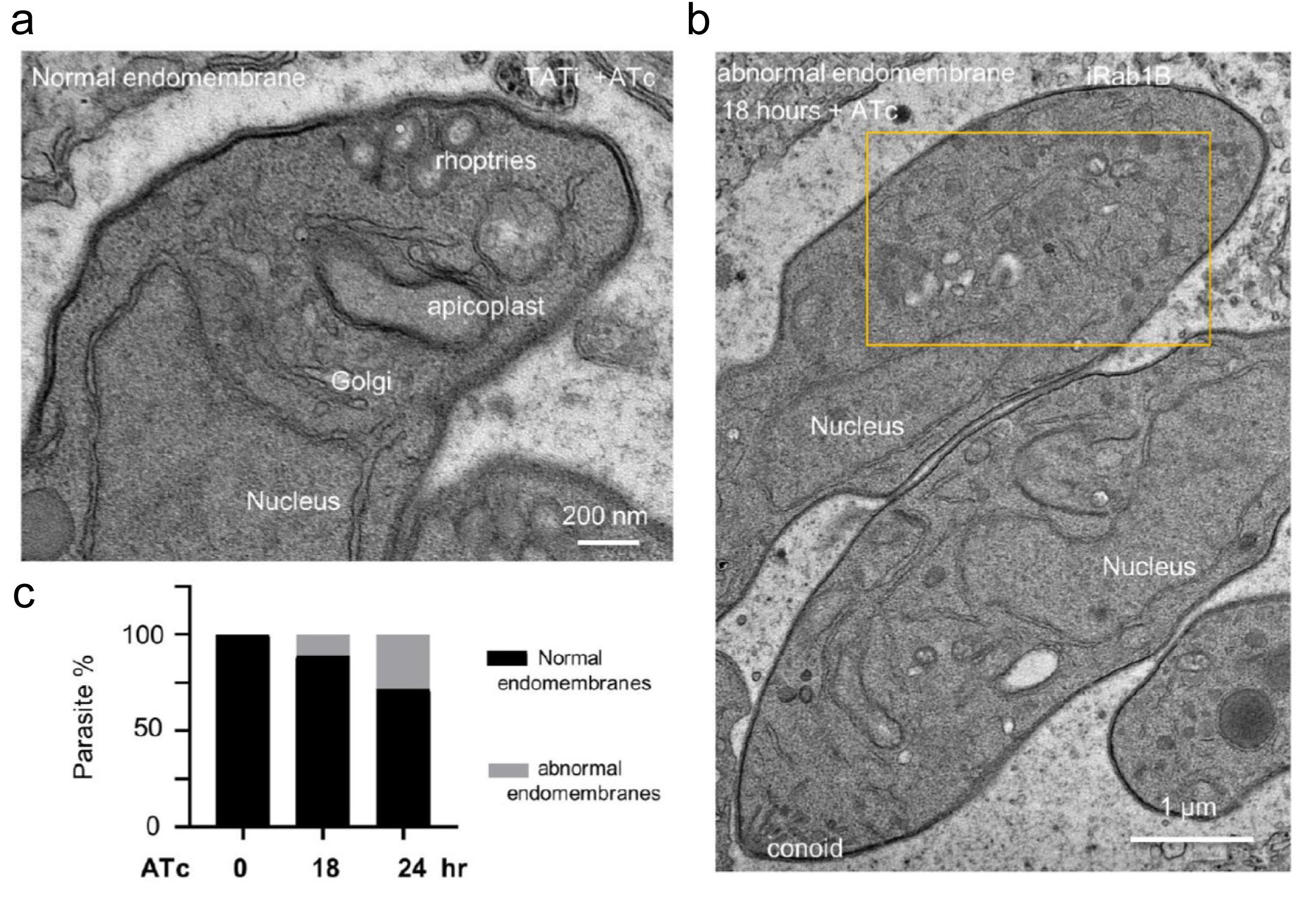

Supplement: Supplementary file 1 — Additional file 1. Depletion of Rab1B caused disorganized endomembrane in parasites. The iRab1B parasites were grown in ATc for 18 and 24 h, followed by fixation, embedding and slicing for transmission electron microscopy (TEM). Normal endomembranes were observed in the TATi line (a), while defects of the endomembrane were observed in the iRab1B induced by ATc for 18h (b). The parasites with normal or abnormal endomembranes were quantified in the TATi and iRab1B lines when Rab1b was treated with ATc for 0, 18 and 24 h, respectively (c). Scale bar = 200 nm. [file 13071_2023_6030_MOESM1_ESM.tif]

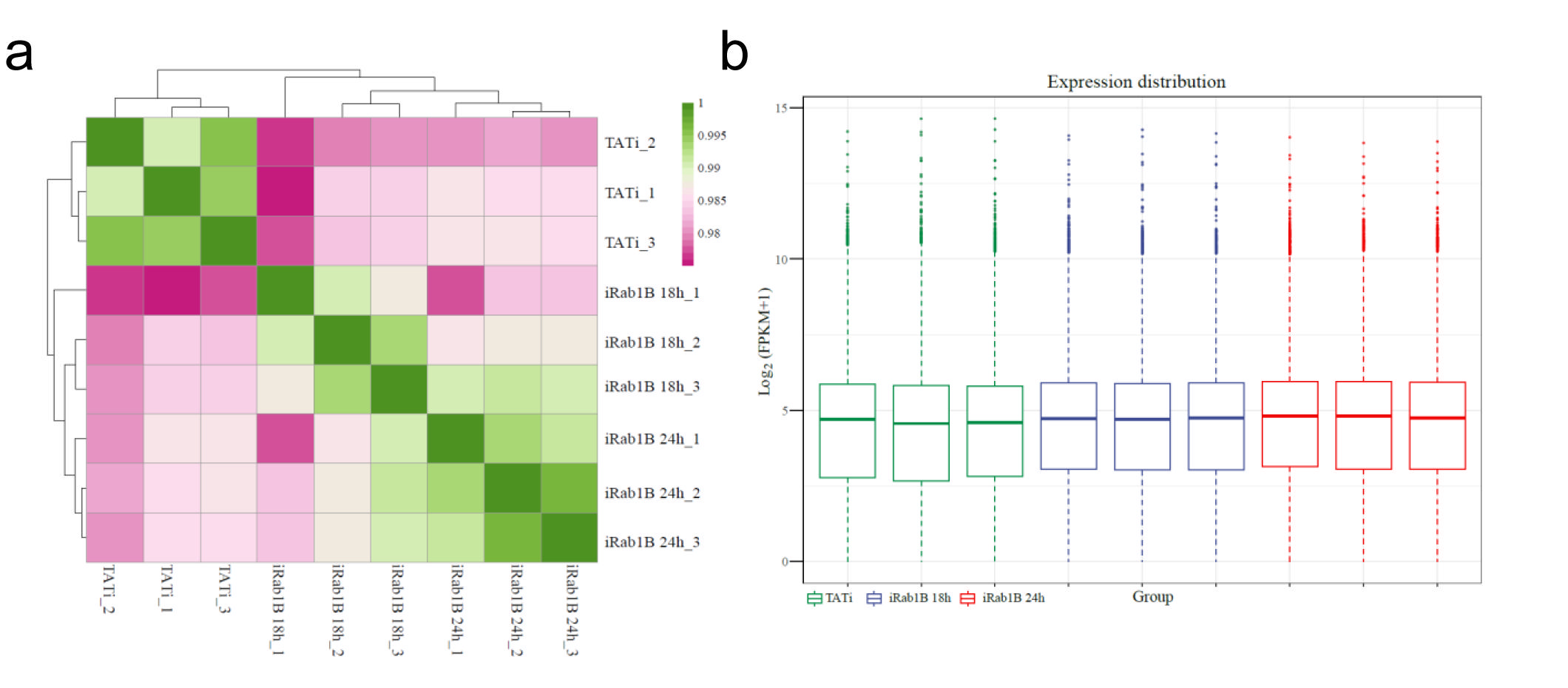

Supplement: Supplementary file 3 — Additional file 3. Global view of transcriptomic expression profiles during TgRab1B degradation. (a) Correlation heatmap analysis showing the relationship in the samples of the control and other two parasite lines. (b) Boxplot showing the FPKM-normalized expression distribution in different groups. [file 13071_2023_6030_MOESM3_ESM.tif]
